# Relapsed Graves’ disease: should continuous low-dose methimazole become a preferred strategy?

**DOI:** 10.20945/2359-4292-2026-0046

**Published:** 2026-04-01

**Authors:** Célia Regina Nogueira

**Affiliations:** 1 Departamento de Medicina Interna, Faculdade de Medicina de Botucatu, Universidade Estadual Paulista (Unesp), Botucatu, SP, Brasil

The optimal management of relapsed Graves’ disease (GD) remains one of the most debated issues in contemporary thyroidology. Although GD is the most common cause of hyperthyroidism in iodine-sufficient regions, therapeutic decision-making after relapse following an initial course of antithyroid drug (ATD) therapy continues to pose important clinical challenges ^(1)^. Traditionally, radioiodine therapy (RAI) and thyroidectomy have been considered definitive treatment strategies. However, accumulating evidence over the past two decades supports prolonged ATD therapy as a safe and effective long-term alternative in selected patients ^(2-5)^.

Relapse after discontinuation of ATD therapy occurs in approximately 40%-60% of patients treated for 12-18 months, emphasizing the need for individualized approaches beyond conventional treatment algorithms ^(2,3)^. In this context, defining the most appropriate management strategy after recurrence represents a critical step toward optimizing long-term outcomes.

In this issue of the *Archives of Endocrinology and Metabolism*, Carlini and cols. ^(6)^ address this clinically relevant dilemma by comparing three therapeutic approaches in patients with relapsed GD: continuous low-dose methimazole, a second course of methimazole until TRAb becomes negative, and RAI followed by levothyroxine replacement. Their study provides valuable real-world evidence that contributes meaningfully to the evolving management paradigm for recurrent disease.

One of the most important observations of the study is the higher proportion of time spent in euthyroidism among patients receiving continuous low-dose methimazole compared with those treated with either RAI or a second ATD course. This finding has clear clinical relevance, since maintaining stable thyroid function represents a central therapeutic objective in GD management. Even mild thyroid dysfunction has been associated with increased cardiovascular risk, including atrial fibrillation, stroke, and mortality, reinforcing the importance of sustained biochemical stability ^(7)^.

These results further support the concept that prolonged ATD therapy should no longer be viewed exclusively as a temporary strategy before definitive treatment but rather as a legitimate long-term therapeutic option in appropriately selected patients. Historically, ATDs were mainly considered a bridge to ablation or surgery. However, this perspective has progressively evolved as long-term observational studies and randomized clinical trials demonstrated that extended methimazole therapy is associated with higher remission rates and reduced recurrence risk without relevant safety concerns ^(4,5,8,9)^. Importantly, the safety profile of long-term low-dose methimazole has been consistently confirmed in systematic reviews showing that severe adverse effects are rare and occur predominantly during the initial months of treatment and at higher drug doses ^(4)^. These findings have contributed to gradual changes in clinical practice patterns, particularly in Europe and Asia, where prolonged ATD therapy is increasingly accepted as a definitive therapeutic strategy in selected patients ^(2,5)^.

Another relevant contribution of the study by Carlini and cols. ^(6)^ concerns thyroid eye disease outcomes. The absence of differences among treatment groups is clinically reassuring, especially because concerns regarding orbitopathy progression frequently influence treatment selection after relapse. Current evidence indicates that long-term ATD therapy represents a safe alternative in patients with mild orbitopathy and may be preferable to RAI in selected clinical scenarios ^(10)^.

Similarly, the lack of differences in quality of life across treatment strategies highlights an essential aspect of GD management. Quality of life impairment is a major determinant of long-term disease burden and frequently persists despite biochemical control. However, available evidence suggests that quality of life is more closely related to thyroid functional status than to treatment modality itself, reinforcing that maintaining euthyroidism remains the primary therapeutic goal ^(11)^.

An additional strength of the study is the long duration of follow-up, exceeding six years in all treatment groups. Such extended observation provides valuable insight into real-world treatment effectiveness and supports the relevance of evaluating long-term biochemical control rather than short-term remission alone. In daily clinical practice, sustained euthyroidism over time is often more meaningful than early treatment response.

The study also raises an important consideration regarding treatment duration after relapse. Patients who achieved remission following a second course of methimazole had longer treatment exposure than those who relapsed, reinforcing previous evidence that remission probability increases with prolonged ATD therapy ^(8,9)^. These findings support a more flexible approach in defining treatment duration rather than adhering to fixed therapeutic intervals.

Another noteworthy aspect concerns the role of TRAb measurement in guiding treatment discontinuation. Although TRAb negativity is frequently interpreted as a marker of immunological remission, the present results suggest that it may not reliably predict sustained clinical remission after therapy withdrawal. This observation highlights the complexity of autoimmune regulation in GD and underscores the limitations of relying exclusively on antibody status to guide therapeutic decisions ^(2,3)^.

The implications of these findings extend beyond individual patient management. In regions where access to definitive therapies may be limited or delayed, long-term pharmacological treatment represents a particularly valuable strategy. In Latin America, where variability in healthcare access persists across different settings, the demonstration that prolonged low-dose methimazole is both safe and effective supports a more flexible and individualized therapeutic approach adapted to regional realities.

Taken together, the results presented by Carlini and cols. ^(6)^ add important evidence supporting continuous low-dose methimazole as a rational long-term strategy for patients with relapsed GD. Rather than representing merely an alternative to definitive therapies, prolonged ATD treatment may in selected cases become a preferred option, particularly for patients seeking to avoid radioiodine exposure or surgery while maintaining stable thyroid function ^(12)^. These findings support a progressive shift toward a more individualized and conservative long-term management strategy for patients with relapsed Graves’ disease.

## Data Availability

datasets related to this article will be avail-able upon request to the corresponding author.

## References

[1] Smith TJ, Hegedüs L. (2016). Graves’ Disease. N Engl J Med.

[2] Kahaly GJ, Bartalena L, Hegedüs L, Leenhardt L, Poppe K, Pearce SH. (2018). 2018 European Thyroid Association Guideline for the Management of
Graves’ Hyperthyroidism. Eur Thyroid J.

[3] Ross DS, Burch HB, Cooper DS, Greenlee MC, Laurberg P, Maia AL (2016). American Thyroid Association Guidelines for Diagnosis and
Management of Hyperthyroidism and Other Causes of
Thyrotoxicosis. Thyroid.

[4] Azizi F, Malboosbaf R. (2019). Safety of long-term antithyroid drug treatment? A systematic
review. J Endocrinol Invest.

[5] Laurberg P, Berman DC, Andersen S, Bülow Pedersen I. (2011). Sustained control of Graves’ hyperthyroidism during long-term
low-dose antithyroid drug therapy of patients with severe Graves’
orbitopathy. Thyroid.

[6] Carlini JA, Santos RB, Perini N, Romaldini JH, Villagelin D. (2026). Treatment outcomes in patients with relapsed Graves’
disease. Arch Endocrinol Metab.

[7] Chaker L, Cooper DS, Walsh JP, Peeters RP. (2024). Hyperthyroidism. Lancet.

[8] Azizi F, Amouzegar A, Tohidi M, Hedayati M, Khalili D, Cheraghi L (2019). Increased Remission Rates After Long-Term Methimazole Therapy in
Patients with Graves’ Disease: Results of a Randomized Clinical
Trial. Thyroid.

[9] Park SY, Kim BH, Kim M, Hong AR, Park J, Park H (2021). The longer the antithyroid drug is used, the lower the relapse
rate in Graves’ disease: a retrospective multicenter cohort study in
Korea. Endocrine.

[10] Burch HB, Perros P, Bednarczuk T, Cooper DS, Dolman PJ, Leung AM (2022). Management of Thyroid Eye Disease: A Consensus Statement by the
American Thyroid Association and the European Thyroid
Association. Thyroid.

[11] Taylor PN, Medici MM, Hubalewska-Dydejczyk A, Boelaert K. (2024). Hypothyroidism. Lancet.

[12] Villagelin D, Romaldini JH, Santos RB, Milkos AB, Ward LS. (2015). Outcomes in Relapsed Graves’ Disease Patients Following
Radioiodine or Prolonged Low Dose of Methimazole Treatment. Thyroid.

